# Multifaceted roles of SARM1 in axon degeneration and signaling

**DOI:** 10.3389/fncel.2022.958900

**Published:** 2022-08-25

**Authors:** Thomas J. Waller, Catherine A. Collins

**Affiliations:** ^1^Department of Molecular, Cellular, and Developmental Biology, University of Michigan, Ann Arbor, MI, United States; ^2^Department of Neurosciences, Case Western Reserve University, Cleveland, OH, United States

**Keywords:** injury signaling, Nicotinamide Adenine Dinucleotide (NAD), MAP Kinase, axonal transport, axon degeneration, SARM1, TIR domain

## Abstract

Axons are considered to be particularly vulnerable components of the nervous system; impairments to a neuron’s axon leads to an effective silencing of a neuron’s ability to communicate with other cells. Nervous systems have therefore evolved plasticity mechanisms for adapting to axonal damage. These include acute mechanisms that promote the degeneration and clearance of damaged axons and, in some cases, the initiation of new axonal growth and synapse formation to rebuild lost connections. Here we review how these diverse processes are influenced by the therapeutically targetable enzyme SARM1. SARM1 catalyzes the breakdown of NAD+, which, when unmitigated, can lead to rundown of this essential metabolite and axonal degeneration. SARM1’s enzymatic activity also triggers the activation of downstream signaling pathways, which manifest numerous functions for SARM1 in development, innate immunity and responses to injury. Here we will consider the multiple intersections between SARM1 and the injury signaling pathways that coordinate cellular adaptations to nervous system damage.

## Introduction

Axons enable neural communication over great distances in the nervous system. Tuned to the vulnerability of long axons, decades of studies (including work by Ramon y Cajal and Augustus Waller in prior centuries) have showcased the plasticity of the nervous system through the varied responses made by individual cells adapting to nervous system damage. These responses include the ability to regrow damaged axons, to repair lost connections and/or sprout new axonal branches. Also critical for nervous system plasticity is the degeneration and clearance of the distal axon “stump” which has lost connectivity with the cell body.

Unless the refusion of axons can occur [which occurs robustly in *C. elegans* (Neumann et al., 2011)], the distal stump has no clear function in its original circuit. Despite this, continued existence of the severed axon may be enabled by localized protein synthesis, energy supply from axonally localized mitochondria (and other organelles), and support from local glia.

While it is logical that the stump should ultimately degenerate from the lack of new organelles or newly expressed proteins transported from the cell body, there is increasing evidence that the mechanism by which most axons degenerate is not simply a passive rundown of cellular function. Rather, axons degenerate through an active and regulated “self-destruction” pathway akin to apoptosis, but involving distinct cellular machinery. A key component of this machinery is the sterile alpha TIR motif containing protein SARM1, whose enzymatic activity drives the loss of NAD^+^, a central electron carrier. This enzymatic activity is essential for SARM1’s ability to drive axonal degeneration. SARM1 has also been shown to function as a regulator of intracellular signaling pathways, which can intersect in multiple ways to drive degeneration. This review focuses on these points of intersection, which help to inform a global, albeit complex view, of how this therapeutically targetable enzyme works to coordinate responses to nervous system damage in multiple cell types.

## SARM1-regulated signaling mediates diverse functions, including responses to nervous system injury

First we review some of the multiple known functions of SARM1 in different cell types. SARM1 is broadly expressed in many different tissues, and within the nervous system it is expressed within glial and immune cells as well as neurons (Hu et al., 2021). Prior to the discovery of SARM1’s best-known role in axonal degeneration, studies in multiple model organisms have identified SARM1 as an upstream regulator of MAP (mitogen activated kinase) Kinase signaling (Liberati et al., 2004; Chuang and Bargmann, 2005; Kim et al., 2007; Chen et al., 2011). Its homolog in *C. elegans* was first discovered for its role in regulating left-right asymmetric cell fate choices between two synaptically connected olfactory neurons (Chuang and Bargmann, 2005; Inoue et al., 2013; Gerdts et al., 2016). This choice is driven by MAP Kinase signaling downstream of the Apoptosis signal-regulating kinase (ASK1), whose activation and localization at synapses requires SARM1’s homolog TIR-1 (Chuang and Bargmann, 2005; Inoue et al., 2013; Gerdts et al., 2016). Additional roles for SARM1-gated MAP Kinase signaling include regulation of neuronal cytoskeleton and neurite structure (Kim et al., 2007; McLaughlin et al., 2016; Izadifar et al., 2021), and the clearance of apoptotic cells by glia (McLaughlin et al., 2019). Both SARM1 and ASK1 regulate Toll-like receptor (TLR) signaling, a key signaling arm of innate immunity (Liu et al., 2014; Matsuzawa, 2017), and act *via* JNK to promote “forgetting” in *C. elegans* olfactory neurons (Inoue et al., 2013). Loss of SARM1 also leads to impairments in synaptic plasticity (Lin et al., 2014) and loss of parvalbumin neurons associated with autism-like behaviors (Xiang et al., 2022).

**FIGURE 1:**
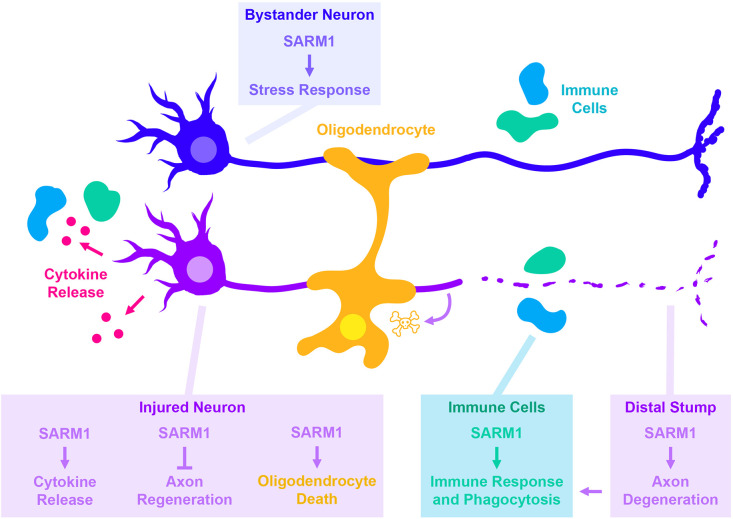
Cell autonomous and non-cell autonomous roles for SARM1 in responses to axonal injury. Each box indicates the cell type SARM1 is acting within, with text color indicating the cell type being impacted by SARM1’s function (same colors indicate cell autonomous, different colors indicate non-autonomous). SARM1’s cell autonomous roles in injured neurons are shown in magenta. In addition to its most well-known for its role in promoting degeneration of the distal stump, SARM1 is also required for additional responses made by injured neurons. These include the JNK- and c-Jun-dependent release of cytokines (Wang et al., 2018), the ability of neurons to regenerate in *C. elegans* (Julian and Byrne, 2020), and in promoting death of oligodendrocytes in a glaucoma model (Ko et al., 2020). SARM1 also functions within uninjured cells that participate in responses to injury, including uninjured “bystander” neurons (dark blue blue) (Hsu et al., 2021), and immune cells that react to the damage (light blue) (McLaughlin et al., 2019).

Several of SARM1’s signaling functions are engaged following axonal injury. These functions (cartooned in Figure 1) are particularly interesting to consider as they tie together multiple responses in different cell types to axonal damage. First, SARM1 functions within injured neurons (colored violet in Figure 1) to regulate several cell autonomous responses to axonal damage. These include SARM1’s most well-known function in promoting Wallerian degeneration of the distal axon stump (Osterloh et al., 2012). Another cell autonomous response is the triggering of cytokine production and release from axotomized DRG neurons following sciatic nerve injury, which is mediated by Jun N-terminal kinase signaling (an arm of MAP kinase signaling) (Wang et al., 2018). This places Sarm1 upstream of an important inflammatory response to nerve damage, relevant for pain and also for the ability of axons to regenerate (Jessen and Mirsky, 2016; Sommer et al., 2018). In contrast to the degeneration of axons severed from the cell body, this response involves changes in gene expression, hence the cell body. A third cell autonomous injury response gated by Sarm1 was recently described in axotomized *C. elegans* motor axons, where Sarm1 inhibits the ability of injured axons to initiate regenerative axon growth (Julian and Byrne, 2020). This response requires the ASK1 kinase and a p38-mediated arm of MAP Kinase signaling.

Events downstream of SARM1 activation also trigger responses in other cells. For example, Wallerian degeneration leads to the generation of axonal debris, which contributes to the initiation and recruitment of immune response. This immune response is hypothesized to be important for axonal regeneration in the peripheral nervous system; supporting this idea, multiple studies have noted that axonal regeneration in the peripheral nervous system is impaired when Wallerian degeneration is inhibited (Lunn et al., 1989; Bisby and Chen, 1990; Brown et al., 1994, 1991; McKerracher et al., 1994; Mukhopadhyay et al., 1994; Schäfer et al., 1996; Rotshenker, 2011). Another example of non-cell autonomous roles for SARM1 was recently shown in a model of glaucoma, where necroptosis signaling leads to the activation of SARM1 (Ko et al., 2020). In this model, knockout of SARM1 not only rescules axonal degeneration, but also the death of neighboring oligodendrocytes, which may be a consequence of the axonal degeneration.

In addition to the above roles in injured neurons, SARM1-regulated signaling also acts in other **non-injured** cells to regulate responses to injury (Figure 1). Hsu et al. (2021) documented a role for *Drosophila* SARM1 in slowing axonal transport in non-injured “bystander” axons. Signaling downstream of SARM1 also regulates the phagocytic capacity of glial cells during *Drosophila* development (McLaughlin et al., 2019), which may potentially be relevant for their ability to clear debris following injury.

## The SARM1 enzyme

While TIR domains typically mediate protein-protein interactions with other TIR domains to scaffold intramolecular complexes, SARM1’s TIR domains have potent enzymatic activity (Gerdts et al., 2015). In its activated form, the SARM1 enzyme cleaves Nicotinamide Adenine Dinucleotide (NAD^+^) into Nicotinamide Mononucleotide (NAM) and cyclic (and non-cyclic) ADP-Ribose (cADPR) (Essuman et al., 2017). The rundown of NAD^+^, a key electron carrier for cellular metabolism, is expected to lead to metabolic catastrophe due to failure of NAD-dependent oxidative reactions and eventual ATP synthesis (Zhao et al., 2019; Figley et al., 2021). SARM1 is required and responsible for axon degeneration under a variety of stress and disease conditions (Coleman and Höke, 2020; Figley and DiAntonio, 2020; Sarkar et al., 2021), and also serves as an executioner of a form of cell death named “sarmoptosis” (reviewed in Fricker et al., 2018). SARM1-mediated neuron death can be triggered by mitochondrial toxins (Summers et al., 2014), and TLR signaling (Mukherjee et al., 2015, 2013). It has also been documented in non-neuronal cells including as a form of programmed cell death to terminate activated T-cells (Panneerselvam et al., 2013).

In addition to NAD^+^ loss, the products of SARM1’s enzymatic reaction may also carry out additional functions, either to facilitate degeneration or additional roles in cellular signaling. Cyclic ADPR, a robustly measurable product of SARM1 enzymatic activity, is a known regulator of the ER Ryanodine receptor and TRPM2 calcium channels (Galione and Ruas, 2005; Galione and Chuang, 2020). This function makes cADPR a prime candidate to mediate intracellular signaling, as well as calcium influx. Of note, the final stages of axonal degeneration are closely accompanied by an increase in intracellular calcium (Geffen and Hughes, 1972; Schlaepfer and Bunge, 1973; Schlaepfer, 1974; Schlaepfer and Hasler, 1979; Mishra et al., 2013; Ko et al., 2021). While some findings did not detect a role for cADPR in Wallerian degeneration of injured axons *in vitro* (Sasaki et al., 2020), recent work has shown a role for cADPR in triggering calcium flux and degeneration in a model of chemotherapy-induced peripheral neuropathy (Li et al., 2021). Hence cADPR may also contribute to the degenerative outcome of SARM1 activation.

Beyond the traditional breakdown of NAD^+^ into NAM and cADPR, recent work has shown that the SARM1 enzyme can carry out multiple base exchange reactions (Angeletti et al., 2022). This includes base exchanges between NAD (and NADP) and the pyrimidines 3-acetylpyridine, vacour, and nicotinic acid. In addition to being a point of vulnerability to neurotoxins (Loreto et al., 2021; Wu et al., 2021), the wide range of reactants and products may enable SARM1 to influence multiple signaling pathways and cellular responses.

Is SARM1’s enzymatic activity an essential property of its signaling mechanism? Structure-functions studies indicate that its TIR domains are essential (Chuang and Bargmann, 2005; Gerdts et al., 2015; Herrmann et al., 2022), and recent studies in Drosophila found that dSarm’s enzymatic activity tracks with its signaling functions (Brace et al., 2022; Herrmann et al., 2022). However, Herrmann et al. (2022) also found some differences: axonal degeneration requires additional domains of SARM1 that are dispensable for its signaling functions. Hence Sarm1 may regulate degeneration and signaling through distinct biochemical states.

## Regulation of SARM1

Since unmitigated SARM1 enzymatic activity can drive cellular degeneration, SARM1’s enzymatic activity is tightly controlled in healthy cells. The catalytic domain of the enzyme is comprised of an octomeric ring formed by the oligomerization of TIR domains from multiple subunits (Bratkowski et al., 2020; Jiang et al., 2020; Sporny et al., 2020). Under normal conditions, the TIR domains are held in an inactive state through intramolecular interactions with SARM1’s ARM domain. The ARM domain contains an allosteric binding site for NAD + and NMN, which either stabilize (for NAD +) or inhibit (for NMN) the inhibitory interaction. Strikingly, the allosteric regulation provides a compelling explanation for the long-held observation that axonal degeneration and SARM1 activation are strongly inhibited by the NAD + biosynthesis enzyme NMNAT (NAM Adenylyl Transferase). NMNAT can regulate SARM1 through its enzymatic activity: it consumes SARM1’s activator NMN to produce NAD +, which inhibits SARM1.

**FIGURE 2:**
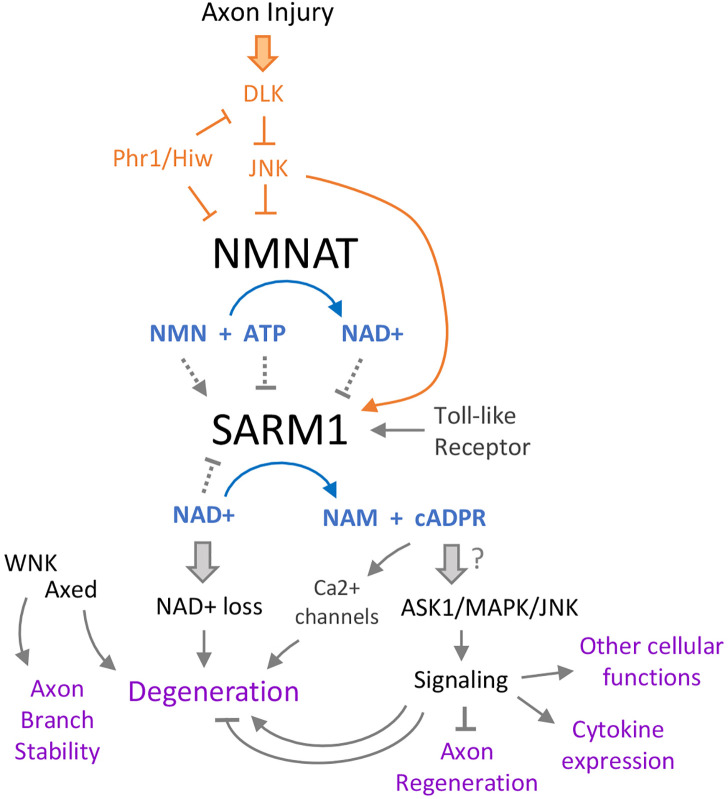
Intertwining relationships between SARM1 and axonal damage signaling. Multiple pathways converge on SARM1, which itself has several effects. Following axon injury, the Duel-leucine kinase (DLK) initiates a kinase cascade that both promotes SARM1 activation and turnover of the NAD^+^ biosynthetic enzyme NMNAT. Loss of NMNAT further promotes activation of SARM1 *via* a shift in the ratio of NAD^+^ to its precursor NMN, both of which are allosteric regulators of SARM1. SARM1’s catalytic state is therefore potently tuned by the function of the NMNAT, which is a critical protective factor in axons and neurons (Gilley and Coleman, 2010). This regulation also enables a feed-forward mechanism of SARM1 activation in injured axons and cells that lack NMNAT function, since once SARM1 is activated it can promote its own further activation by lowering NAD^+^ levels. Downstream of SARM1, loss of NAD^+^ leads to axon breakdown *via* an unclear mechanism, but is thought to be tied to rapid ATP loss and catastrophic metabolic rundown. One of SARM1’s products, cyclic ADP-ribose (cADPR) has increasingly gained prominence in the model of SARM1’s signaling. Acting on multiple calcium channels, cADPR promotes degeneration *via* Ca^2+^ flux in the axon. There have also been links between this metabolite and SARM1’s protein signaling activities, which act through several MAP kinases to promote axon regeneration (from the axon segment connected to the cell body), cytokine expression, neurite development, and other functions.

By inhibiting SARM1 activation, NMNAT enzymes are critical “survival” factors for cells. The levels and localization of the cytoplasmic isoform, NMNAT2, are highly regulated in neurons. Multiple mechanisms are known to regulate the stability, turnover and/or localization of NMNAT enzymes, and some of these are also linked to or signaling pathways that become activated by axonal damage (Figure 2). Most notably, this includes JNK signaling, which regulates NMNAT levels (Milde et al., 2013; Walker et al., 2017), and evolutionarily conserved E3 ubiquitin ligase, Phr1 (Xiong et al., 2012; Babetto et al., 2013).

It is expected that similarly to NMNAT, there should be additional mechanisms that directly regulate SARM1. SARM1 is phosphorylated, which has been shown to stimulate its NAD + cleavage activity (Murata et al., 2018; Xue et al., 2021). Recent work suggests that SARM1’s activation state tracks with a phase transition; in *C. elegans* intestinal epithelial cells, fluorescently labeled SARM1 ortholog (TIR-1) could be observed to coalesce into visible puncta during conditions of stress that activate its downstream signaling (Loring et al., 2021). The phase transition and activation of downstream signaling can be triggered in genetic conditions that result in cholesterol scarcity, suggesting a cellular mechanism for how conditions of cellular stress can lead to SARM1 activation (Peterson et al., 2022).

SARM1 activation also occurs in additional conditions of high translational relevance, which include treatment with chemotherapy agents that induce neuropathy (Bosanac et al., 2021), and TNF-alpha triggered inflammation in an inflammatory model of glaucoma (Ko et al., 2020). Importantly, SARM1 inhibition rescues axon function and slows both axon degeneration and cell death. In the Ko et al. (2020) study, the induction of necroptosis signaling *via* Mixed Lineage Kinase domain-Like pseudokinase (MLKL) was shown to be responsible for SARM1 activation, and subsequent SARM1-mediated axonal degeneration. This SARM1 activation can be rescued by inhibiting MAP Kinase/JNK signaling (Ko et al., 2020), which, as discussed above, can regulate the levels NMNAT2 (Walker et al., 2017). Therefore SARM1 activation during necroptosis signaling may converge with its regulation by NAD^+^ /NMN. To summarize, we know thus far of multiple mechanisms that control SARM1’s enzymatic activity, but most converge on NMNAT, and MAP Kinase signaling pathways activated by axonal damage.

## Axonal damage signaling

Axonal damage triggers the activation of multiple signaling pathways within the damaged neurons, which influence cellular responses ranging from neuronal death to the initiation of new axonal growth for facilitating repair and/or the ability to form alternative synaptic connections. Stress-associated signaling *via* the dileucine zipper kinase DLK and downstream Jun N-terminal Kinases (JNKs) are known to be activated from multiple aspects of axonal injury response (Asghari Adib et al., 2018; Jin and Zheng, 2019). These kinases have been observed appearing within damaged axons at short time periods after nerve injury (Cavalli et al., 2005; Lindwall and Kanje, 2005; Yang et al., 2015).

Multiple observations have documented an intimate web of interactions between axonal damage signaling and axonal degeneration machinery (Figure 2). First, several studies have pointed to roles for JNK signaling in the execution of distal stump degeneration. Acute application of JNK inhibitors to axomized DRG neurons in culture concomitant with injury is capable of delaying the onset of degeneration (Miller et al), suggesting a local and acute role for JNK in axotomized axons. One pro-degenerative role for JNK is promoting the turnover of NMNAT2, as discussed above (Walker et al., 2017). In addition, all 3 JNK kinase isoforms are capable of phosphorylating SARM1, and phosphorylation of SARM1 activates its enzymatic activity and subsequent neurodegeneration (Murata et al., 2018; Xue et al., 2021). While the above studies suggest upstream regulatory roles for JNK signaling in driving the activation of SARM1, other studies have suggested a converse relationship where JNK signaling activation in damaged axons may be triggered downstream of SARM1 activation (Yang et al., 2015; Wang et al., 2018).

It is also plausible that signaling gated by SARM1 acts together with its enzymatic activity to drive degeneration. One study has posited a role for JNK downstream of SARM1, since genetic disruption of all 3 JNK isoforms (or both MKK4/7) could inhibit degeneration caused by ectopic activation of SARM1’s TIR domains (Yang et al., 2015). The dual upstream and downstream relationships of JNK signaling with SARM1 (and vice versa) suggests the possibility that they cycle together in a feed-forward relationship, acting to both promote axonal degeneration and detect the presence, likelihood, or possibility of axonal degeneration following SARM1 activation. These relationships suggest intimate links between axonal damage signaling and SARM1 that we have yet to understand on a molecular level (Figley et al., 2021; Waller and Collins, 2021).

One more set of twists in the intertwined relationship of JNK signaling with SARM1 come from observations in invertebrates that JNK signaling activation following axonal damage can have protective functions in neurons. This includes the inhibition of degeneration in both axons and dendrites (Xiong and Collins, 2012; Hao et al., 2019), which contrasts with the pro-degenerative role described above for JNK in mammalian axons. The mechanism for this protective role is not yet clear. However, it appears to require the transcription factor Fos and may therefore be mediated by downstream changes in gene expression. This could possibly promote multiple changes to axonal metabolic mechanisms or cytoskeleton that influence the propensity of axons to degenerate. While it is plausible that the Fos-dependent pathway could influence the expression level or localization of NMNAT enzyme isoforms(s) (Chen et al., 2016), a recent study has described an NMNAT-independent form of JNK-mediated protective signaling (Hao et al., 2019).

## Potential new players in SARM1 signaling mechanisms

Recent work has brought to light some additional players in NMNAT-SARM1 signaling. One intriguing player is Axed, short for “Axundead,” which was discovered in a forward genetic screen in *Drosophila* (Neukomm et al., 2017). Similar to mutants in dSarm, neurons lacking *axed* function fail to undergo Wallerian degeneration. Remarkably, *axed* mutants inhibit axonal degeneration even in conditions that are expected to result in unmitigated NADase enzymatic activity by dSarm. This includes deletion of NMNAT, which normally leads to axonal and neuronal degeneration, but is rescued by mutation of Axed. From these striking genetic data it is proposed that Axed promotes axonal degeneration downstream of SARM1’s enzymatic activity.

How Axed may do this remains mysterious. It contains a BTB domain, which could participate in scaffolding signaling interactions, but has no known enzymatic activity of its own. Axed protein is abundantly localized near presynaptic terminals in *Drosophila* motoneurons. There are four orthologs of Axed in mammals: BTBD1, BTBD2, BTBD3, and BTBD6 (Neukomm et al., 2017). Potential functional overlap or redundancy has made it hard to determine whether any of these proteins functionally analogously to Axed in mammals.

Another intriguing new set of players are the Lysine Deficient Protein WNK (abbreviated for “with-no-lysine K”) kinases. WNK kinases are broadly expressed, best known for roles in the regulation of ion transport and blood pressure regulation (Murthy et al., 2017). However a recent study, also resulting from a genetic screen in *Drosophila*, identified developmental roles for dWnk in stabilizing axonal branches, in addition to regulating branching during neurite outgrowth (Izadifar et al., 2021). Knockdown of dWnk leads to both axon branching defects and early onset degeneration. This degeneration is dependent on Axed and is rescued by dNMNAT activity (similarly to SARM1-mediated Wallerian degeneration). In axon maintenance, dWnk promotes dNMNAT activity while negatively regulating both Axed and dSarm based on genetic epistasis data. Therefore WNK appears to be an important regulator of at least two functions for signaling surrounding SARM1. Future study of the cellular mechanism of its interactions with SARM1 may provide important insights into SARM1’s functions in signaling as well as degeneration.

## Conclusion

The SARM1 NADase enzyme is an attractive druggable target for intervening neuropathies and a variety of neurodegenerative diseases. SARM1’s enzymatic activity drives the degeneration of axons and, in addition, regulates signaling pathways that influence development, inflammation, and multiple responses to axonal injury. Further consideration of SARM1 as an enzymatic target requires a deeper understanding of SARM1’s roles in development and the crosstalk of SARM1’s multiple functions in the context of nervous system injury.

## Author contributions

TW and CC worked in collaboration to carry out the conception and writing and revising of this review. Both authors contributed to the article and approved the submitted version.
